# Neologistic jargon aphasia and agraphia in primary progressive aphasia

**DOI:** 10.1016/j.jns.2008.10.014

**Published:** 2009-02-15

**Authors:** Jonathan D. Rohrer, Martin N. Rossor, Jason D. Warren

**Affiliations:** Dementia Research Centre, Department of Neurodegenerative Disease, Institute of Neurology, University College London, UK

**Keywords:** Jargon aphasia, Primary progressive aphasia, Dementia

## Abstract

The terms ‘jargon aphasia’ and ‘jargon agraphia’ describe the production of incomprehensible language containing frequent phonological, semantic or neologistic errors in speech and writing, respectively. Here we describe two patients with primary progressive aphasia (PPA) who produced neologistic jargon either in speech or writing. We suggest that involvement of the posterior superior temporal–inferior parietal region may lead to a disconnection between stored lexical representations and language output pathways leading to aberrant activation of phonemes in neologistic jargon. Parietal lobe involvement is relatively unusual in PPA, perhaps accounting for the comparative rarity of jargon early in the course of these diseases.

## 1 Introduction

The production of incomprehensible language containing frequent phonemic distortions, semantic errors or neologisms secondary to neurological disease has been termed jargon aphasia (or if writing is affected, jargon agraphia). The production of inappropriate language can be considered in the context of either normal propositional speech or writing, or in the production of single words in the context of naming tasks performed during neuropsychological assessment. Three types of jargon aphasia have been described [1,2]: the production of language which is devoid of content and consists of real words that are inappropriate given the context of the situation (*semantic jargon*); the production of language containing inappropriate words that are nonetheless phonemically-related to what the patient is attempting to convey, and may therefore be either real or non-existent words (*phonemic or phonological jargon*); and the production of language containing non-existent words or true neologisms, which are not phonemically-related to the target (*neologistic jargon*). Patients may have one or more of these types of jargon as part of the same disorder. The occurrence of true ‘abstruse’ neologisms is most common in acute neurological disorders and in particular Wernicke's aphasia.

Analogously, jargon agraphia can comprise semantic jargon, phonological jargon (phonologically-related misspelled words which can be either real words or nonwords) and neologistic jargon [3–6]. Jargon aphasia and agraphia can occur in the same individual but they can also occur in the presence of normal output in the other language channel [4,7]. They are rarely described in the setting of neurodegenerative disease [8,9]. Here we describe two cases of neologistic jargon in primary progressive aphasia (PPA) [10–12]: jargon aphasia in a case of atypical semantic dementia (SD) and jargon agraphia in a case of progressive non-fluent aphasia (PNFA).

## 2 Case reports

### 2.1 Case 1

A 75 year-old right-handed woman presented with a three year history of word-finding difficulties. Her husband had noticed she would frequently use ‘thing’ in place of a more specific word and would confuse words of related meaning (such as ‘door’ for ‘window’). For the previous two years she had also had increased difficulties with arithmetic, writing and spelling. Over the same time period her comprehension of speech had also deteriorated. There had been no significant difficulties with episodic memory and she had never become lost. There were no behavioural symptoms or changes in appetite. There was no family history of dementia. When first assessed she had a fluent aphasia with circumlocutory speech. Repetition for single words was preserved but sentence repetition was impaired. There was bilateral limb apraxia. The general neurological examination was normal. Detailed neuropsychological assessment revealed severe anomia (only able to produce ‘train’ on a simple naming task) and impaired comprehension (13/50 on the British Picture Vocabulary Scale [13]), poor reading skills (2/50 on the National Adult Reading Test [14]) with errors for both irregular and nonwords as well as evidence of parietal dysfunction consisting of dyscalculia (0/24 on the Graded Difficulty Calculation Test [15]), poor spelling and decreased digit span (four digits forwards, unable to repeat two digits backwards.). There was also evidence of executive dysfunction.

Over the next year the patient's speech became more circumlocutory and with increased word-finding difficulties. In addition, abstruse neologisms emerged in her spontaneous speech and she produced neologistic jargon on a simple naming task and when reading (see Table 1). She would produce words that were completely unrelated to the target word (e.g., ‘adepgood’ for ‘spade’). At a further assessment seven months later she continued to produce multiple abstruse neologisms (Table 1). She showed no awareness of the errors she made.

It is difficult to characterise the syndromic diagnosis in this patient. Based on the leading features of fluent, empty speech with profound anomia, loss of word meaning, impaired single word comprehension and surface dyslexia, the case fulfilled modified consensus criteria for SD [10,16]. However, the early development of dominant parietal lobe deficits (dyscalculia, limb apraxia and decreased digit span) is clearly atypical for SD, and suggests that the syndrome here might be more appropriately characterised as the recently described logopenic/phonological aphasia (LPA) variant of PPA [12,17]. While patients with LPA are generally considered to have relatively intact single word comprehension, anomia is often severe and the spectrum of deficits in this syndrome has not been fully defined; it is therefore possible that deficits of single word comprehension may develop in at least a proportion of cases as the syndrome evolves.

#### 2.1.1 Brain imaging

**Fig. 1:**
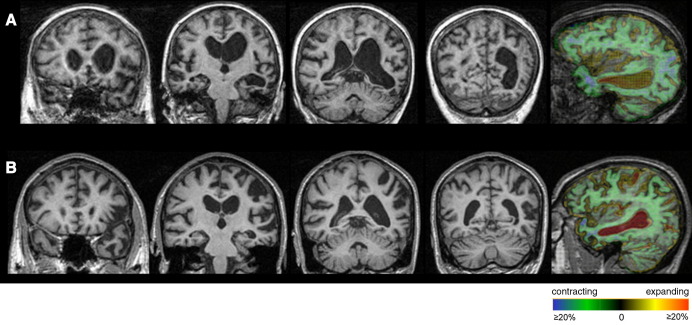
Coronal T1-weighted MR images (with left hemisphere shown on the right of the images) through the frontal, mid-temporal, posterior temporo-parietal and posterior parietal regions and a sagittal MR image through the left temporo-parietal region with a voxel-compression-mapping overlay to show the progression of regional atrophy (degree of volume loss and expansion coded in the colour scale: red represents 20% or greater expansion of voxels and blue represents 20% or greater contraction of voxels.): A) Case 1 — coronal images 5 years after symptom onset; sagittal image shows change over time period 3.5 to 5 years from symptom onset. B) Case 2 — coronal images 4.5 years after symptom onset; sagittal image shows change over time period 3.5 to 4.5 years from symptom onset.

The patient had volumetric brain MRI scans (Fig. 1A) 3.5 and 5 years from symptom onset i.e. pre and post the onset of jargon. Visual inspection of the baseline scan revealed asymmetrical atrophy affecting predominantly the left cerebral hemisphere and, in particular, the temporal lobe and, to a lesser extent, the parietal lobe. There was no antero-posterior gradient of atrophy within the temporal lobe and the superior, middle and inferior temporal lobe gyri were all affected. There was no vascular disease. The pattern of regional atrophy progression between the two scans (i.e. over the period when jargon developed) was assessed using a fluid registration technique producing a voxel compression map, as described previously [18,19]. This showed that progressive atrophy was maximal in the left temporal and inferior parietal lobes (see Fig. 1A), with additional heavy involvement of dorsal prefrontal areas that are likely to be functionally connected with the inferior parietal lobe [20].

### 2.2 Case 2

A 70-year-old right-handed man presented with an eighteen month history of progressive speech production impairment. There were no other cognitive or behavioural symptoms. When first assessed he had a non-fluent aphasia with phonemic paraphasias, agrammatism and poor polysyllabic word and sentence repetition. He also had evidence of a mild motor speech disturbance with hesitancy and effortfulness in articulation. The Mini-Mental State Examination score [21] was 25/30 with points lost on naming, writing and registration. The general neurological examination was normal. There was no family history of degenerative disease. EEG performed at this time showed excess slow activity in the left frontotemporal region but there was preserved alpha rhythm.

Detailed neuropsychological assessment at presentation revealed a verbal IQ of 77 and a performance IQ of 148 on the WAIS-R [22]. Despite the speech production impairment naming was relatively intact at this time scoring between the 75th and 90th percentile on the Graded Naming Test [23]. There was evidence of mild executive dysfunction (Modified Card Sorting Test [24]), mild to moderate impairment of calculation (Graded Difficulty Calculation Test) and decreased digit span (four digits forwards). However, single word comprehension was intact (50–75th percentile on the Synonyms test [25]) as was memory (25th percentile on the Warrington Recognition Memory Test for Words and 75th on the Faces subtest [26]). Visuoperceptual skills were also intact (18/20 on the Object Decision subtest of the VOSP [27]).

Over the next two years the patient's speech production continued to deteriorate and he developed difficulties with speech comprehension. In order to communicate he would write things down but there were frequent grammatical and spelling errors. There was also evidence of impaired calculation although no behavioural abnormalities. When assessed three and a half years after the onset of symptoms there was little spontaneous speech output beyond ‘yes’ and ‘no’. There was evidence of orofacial apraxia although no limb apraxia. Neuropsychological assessment at this time revealed a Raven's matrices equivalent IQ score of 120, intact memory (50–75th percentile on the Camden Pictorial Memory Test [28]) and intact visuoperceptual skills (75–100th percentile on the Object Decision subtest of the VOSP). There was executive dysfunction as previously. In addition there was now evidence of deterioration in single word comprehension, scoring only at the 10th to 25th percentile on the Synonyms test. There was profound anomia: on the Graded Naming Test he was only able to provide written answers to the test (Table 2) with multiple phonological (e.g., ‘squeezers’ for ‘tweezers’) and semantic (e.g., ‘elephant’ for ‘anteater’) errors and evidence of perseveration. On a further writing task he was asked to construct sentences containing a target word: he produced grossly agrammatic and often nonsensical phrases containing semantic errors, though no neologisms.

When assessed one year later he was almost mute. Speech comprehension had further deteriorated, now scoring below the 5th percentile on the Synonyms test. Written responses to the Graded Naming Test (Table 2) contained phonological (e.g., ‘rudii’ for ‘radius’), semantic (e.g., ‘hood’ for ‘cowl’) and perseverative errors as previously. However, these were now accompanied by multiple abstruse neologisms completely unrelated to the target word (e.g. ‘magiff’ for ‘sporran’, ‘gatyss’ for ‘centaur’). He appeared unaware of these errors. He was assessed once more when completely mute a further year later when he scored 8/20 on a subset of the British Picture Vocabulary Scale [13]: although this score is above chance it falls below the 5th percentile. Once again there were multiple abstruse neologisms on the Graded Naming Test with frequent perseverations and illegal letter combinations (e.g. in the neologism IN-KINJCK) (Table 2).

As in Case 1, the syndromic diagnosis in this patient is not clear-cut. Based on the leading features of speech production impairment with agrammatism, sound errors and hesitancy, the clinical presentation here fulfilled modified consensus criteria for PNFA [10,12]. However, other features, in particular the presence of parietal lobe deficits (dyscalculia, decreased digit span) at presentation would be in keeping with the LPA variant.

#### 2.2.1 Brain imaging

The patient had volumetric brain MRI scans (Fig. 1B) 3.5 and 4.5 years from symptom onset, i.e. pre and post the onset of jargon. Visual inspection revealed asymmetrical cerebral atrophy more marked on the left and particularly involving the inferior frontal gyrus and peri-Sylvian region, with extension into the left parietal lobe. There was no vascular disease. As with case 1, the pattern of regional atrophy progression between the two scans (i.e. over the period when jargon developed) was assessed using a fluid registration technique (Fig. 1B). This showed progressive atrophy extending posteriorly surrounding the Sylvian fissure with heavy involvement of the left inferior parietal lobe, in particular the angular gyrus (see Fig. 1B).

## 3 Discussion

Both patients described here developed neologistic jargon in the context of a neurodegenerative disease with a progressive aphasia phenotype. It is of interest to consider why jargon may have developed in these cases. While neologisms are common in aphasias resulting from acute focal brain damage (in particular strokes affecting the posterior superior temporal–inferior parietal region), neologistic jargon has rarely been reported in neurodegenerative disease [8,9]. In particular, it is not mentioned in consensus criteria for frontotemporal lobar degeneration (FTLD) subtypes including PPA [10,11,29] nor in recent reviews of PPA or FTLD [30,31]. The speech of patients with SD often consists of empty, circumlocutory phrases somewhat similar to those produced by patients with stroke aphasias such as transcortical sensory aphasia or Wernicke's aphasia [32,33], however neologisms are rarely reported. One previously described case of SD studied late in the disease course exhibited nonword production on a verbal fluency task although spontaneous neologisms were not described [34]. To our knowledge neologisms have not been described previously as a feature of PNFA or LPA. Both cases here had a clinical syndrome of PPA with additional features that would be atypical for FTLD yet would not fulfil alternative diagnostic categorisations such as Alzheimer's disease (AD). Although Case 1 had clear evidence of severe semantic memory impairment there were also early clinical features of dominant parietal lobe impairment which would not be typical of SD. Moreover, findings on brain imaging were not typical for SD [35] in that there was no anteroposterior gradient of atrophy in the temporal lobes, the left superior temporal gyrus was heavily involved, and atrophy extended posteriorly to involve the left parietal lobe. Case 2 had a diagnosis of PNFA, presenting with classical features of non-fluent speech, agrammatism, phonemic paraphasias and impaired polysyllabic word repetition. Of note, as well as asymmetrical left-sided predominant temporal lobe atrophy, he also had early involvement of the dominant inferior parietal lobe both clinically (dyscalculia) and radiologically with extension of atrophy along the Sylvian fissure. This pattern of atrophy is described in previous cases of PNFA although more often in the presence of a corticobasal degeneration syndrome which Case 2 did not have. The occurrence of neologistic errors in speech may be difficult to interpret in the setting of severe speech production impairment associated with speech apraxia and/or dysarthria. However, Case 2 exhibited clear neologistic errors in written output, demonstrating that such errors represent a true jargon language disturbance in the context of a non-fluent aphasia.

We do not argue that jargon was the only salient feature of the language disturbance in these cases (Case 2, for example, clearly made perseverative errors: see Table 2). Rather, we propose that the less typical finding of jargon in neurodegenerative disease (PPA) may have localising value as a clinical signature of the anatomical pattern of disease spread and may constitute a clinico-anatomical analogue of jargon in acute aphasia. While the histopathological diagnosis in our cases must remain moot, taken together, the clinical and radiological findings are consistent with the concept that involvement of the posterior superior temporal and parietal lobes may modify the phenotype of patients who present with progressive language impairment due to a neurodegenerative disorder (PPA). The parieto-temporal distribution of disease may lead to the appearance of neologistic jargon in a proportion of such cases. In SD and PNFA posterior temporal and parietal lobe involvement is usually a late feature, whereas in LPA these regions are implicated at presentation [17]. Both our cases exhibited features in keeping with a diagnosis of LPA though the presence of semantic impairment in the first case and motor speech impairment in the second case would not be typical of the cases previously described. However, the LPA syndrome has not yet been completely characterised: from the evidence of the present cases, and a priori on anatomical grounds, we propose that detailed analysis of the spoken and written output of patients with LPA may reveal a relatively higher frequency of neologisms than is found in other PPA syndromes. It is noteworthy that detailed analysis of speech errors in patients with AD (in which parietal lobe involvement is typically prominent) reveals a number of similarities with ‘Wernicke's aphasia’ [36], though aphasic disturbances including jargon may be relatively de-emphasised in the face of the more widespread deficits that accompany evolving AD.

Proposed explanations of the core defect in neologistic jargon aphasia include failure of lexical retrieval and impaired monitoring of own speech [37]. The former defect would account for failure to activate the correct item from the lexical store, while the latter defect could account for the frequent observation (as here) that patients with jargon language are frequently unaware of the errors they make. Self-monitoring is a complex neurolinguistic process with a number of elements which may break down in disease [38]. It has been shown that failure of self-monitoring cannot be solely due to impaired speech comprehension and this would be consistent with the observation that most patients with primary progressive language disorders who have impaired comprehension do not produce jargon [37].

Jargon agraphia has been described in association with a number of anatomical lesions including both left and right-sided temporal lobe atrophy [8,9,39]. It often occurs in conjunction with Wernicke's aphasia, consistent with involvement of different language channels as part of the core syndrome. A number of deficits have been proposed to underpin jargon agraphia: these include impaired assembly of graphemes prior to production (the ‘graphemic buffer’), an impaired spelling system, and impaired access to orthographic information. While it is not possible to determine which if any of these deficits is responsible in our Case 2, involvement of the dominant parietal lobe suggests that impaired access to stored orthographic representations is plausible, and might implicate a mechanism analogous to that governing spoken output. Due to the sparsity of spontaneous speech in Case 2, it is not clear to what extent jargon agraphia signifies differential involvement of spoken and written language output pathways in this case. Unlike most reported cases of jargon aphasia and agraphia, neologistic production in Case 2 was at a single word level rather than a sentence level, arguing against a simple compensatory process [6].

Failure of lexical retrieval or self-monitoring in jargon aphasia would not in itself account for the production of novel, meaningless material (neologisms) [6], and the lower frequency of this syndrome in chronic degenerative compared with acute disease states also remains to be explained. Functional disconnection between stored lexical representations and the language output pathways could lead to aberrant or random activation of phonemes in neologistic jargon, due to damage involving a key interface for linking stored lexical templates with verbal output in the posterior superior temporal–inferior parietal region [20]. This would be consistent with the emergence of neologisms in both Wernicke's and conduction aphasia [40]. We would predict that degenerative disease heavily involving this posterior region should also give rise to neologisms. The existence of posterior variants of PPA and their clinical correlates is a major emerging theme in the literature of the progressive aphasias [41]. It will be of interest to establish whether jargon may be relatively more prevalent in patients with the parieto-temporal LPA phenotype, or more specifically, in cases of PPA associated with mutations in the progranulin gene, as early parietal lobe involvement may be more frequent in this subtype [42,43]. The present cases illustrate the importance of longitudinal assessment of language in patients with PPA and other neurodegenerative diseases, and the need for clinicians to remain alert to the emergence of features that may signal particular patterns of disease in the brain and which may therefore ultimately have diagnostic value. A larger prospective study with post mortem correlation would be required to clarify fully the anatomical correlates of this phenomenon and its histopathological associations.

It is likely that involvement of the posterior temporal–inferior parietal region is necessary but not of itself sufficient for the development of jargon in degenerative disease. In addition to the macroscopic distribution of disease, other factors such as the potential for partial functional compensation or reorganisation in progressive disease states and the microscopic distribution of tissue pathology within local cortical networks may influence the development of jargon. The study of patients with jargon may therefore provide insights into the broader and more fundamental issue of the brain mechanisms that underpin phenomenological similarities and divergences between the acute and progressive aphasias [41,44].

## Figures and Tables

**Table 1:** Simple picture naming task and spoken responses from Case 1 (International phonetic alphabet characters in parentheses; Response 1 at 4 years after onset)

	Target	Response 1	Response 2 (+ 7 months)
1	Lobster	Delkwai (dɛlkwɑɪ)	Joon (dʒuːn)
2	Tricycle	Doopid (duːpɪd)	Pekakis (pɛkækɪs)
3	Spade	Adepgood (ædɛpgʊd)	Haygis (heɪgɪs)
4	Owl	Baybeeay (beɪbiːeɪ)	Veeches (viːtʃɛz)
5	Violin	Atepown (eɪtpaʊn)	Joh (jɘʊ)
6	Hippopotamus	Six twenty	Beeap (biːæp)

**Table 2:** Written answers provided for the Graded Naming Test from Case 2 (Response 1 at 3.5 years after onset)

	Target	Response 1	Response 2 (+ 1 year)	Response 3 (+ 2 years)
1	Kangaroo	Kangaroo	Kangoroo	Gown
2	Scarecrow	Scarecrows	Breakfast	Neckor
3	Buoy	Bouys	Bouy	About
4	Thimble	Thimble	Thumb	Next
5	Handcuffs	Handcuffs	Handcuff	Newt-nocket
6	Tweezers	Squeezers	Fistcuff	Newbot
7	Corkscrew	Corkscrews	Squidell	Newbot
8	Sporran	Kilts	Magiff	Newbolt
9	Tassel	Tassle	Gnome	Newbolt
10	Sundial	Timescale	Gnome	Newbolt-brine
11	Chopsticks	Croquets	Forstell	Newbolt
12	Periscope	Periscope	Perspime	Sinks-trinket
13	Boar	Boars	Boar	Basin-misskikiet
14	Blinkers	Baskets	Squid	Binstass
15	Monocle	Moncle	Bonecule	Sinks
16	Turtle	Tortoise	Torquise	Trins-massiness
17	Trampoline	Tamptoise	Bonecule	Misskiten
18	Bellows	Bellows	Fireball	Niss-en
19	Shuttlecock	Tamblecocks	Cockell	Miss-in-teken
20	Anteater	Elephants	No written response	Miss-in-takin
21	Pagoda	Pelicans	No written response	In-takin
22	Radius	Radius	Rudii	No-noken
23	Leotard	Costume	Catuss	In-token-no
24	Mitre	Mitre	Mitre	In token-motoken
25	Yashmak	Masks	Shiel	Juink-
26	Sextant	Sextents	Sextent	In-junk
27	Centaur	M	Gatyss	In-kinjck
28	Cowl	Hoods	Hood	In juink-injuink
29	Tutu	Fluffs	Baysonne	In juink-bosment
30	Retort	Glass	No written response	Jackoo
